# Effect of hybrid rice varieties on growth and development of broilers and ducks

**DOI:** 10.1016/j.aninu.2020.06.004

**Published:** 2020-11-14

**Authors:** Frederick M. LeMieux, Courtney P. Villemarette, Eddie K. Lyons, Thomas H. Shields, Norman German

**Affiliations:** School of Agricultural Sciences, McNeese State University, Lake Charles, LA, 70609, United States

**Keywords:** Hybrid rice, Broiler, Mallard duckling, Mallard duck

## Abstract

Three experiments (Exp. 1, *n* = 144 broilers [Ross × Ross]; Exp. 2, *n* = 118 mallard ducklings [*Anas platyrhynchos*]; and Exp. 3, *n* = 75 mature mallard ducks) were conducted to determine the effects of 3 levels of unmilled hybrid rice on growth performance and organ and gastrointestinal tract development. The dietary treatments were 1) corn-soybean meal (basal), 2) basal + 5% hybrid rice, and 3) basal + 10% hybrid rice for Exp. 1 to 3, respectively. One bird from each pen in Exp. 1 (*n* = 24) and all the birds in Exp. 2 (*n* = 118) and Exp. 3 (*n* = 75) were randomly selected and euthanized to determine linear measurements and organ and gastrointestinal tract weight. In Exp. 1 and 2, birds fed 10% rice experienced slower growth (*P* < 0.05) than birds fed the basal diet. In Exp. 3, the addition of rice did not affect growth performance. Rice addition did not affect organ length or weight (*P* > 0.05) in Exp. 1. However, birds fed 5% rice had significantly increased (*P* < 0.05) pancreas, ileum, and jejunum weights in Exp. 2, and 10% rice significantly increased (*P* < 0.05) liver weight in Exp. 3. The addition of 10% unmilled rice to broiler and duck diets may reduce growth performance.

## 1 Introduction

Rice is a significant part of Louisiana's farm income. In 2018, the gross farm value generated by all rice production was $379.6 million with approximately 436,000 acres planted (LSU AgCenter Staff, 2018). Approximately 59% of the crop was a Clearfield variety or hybrid. In 2018, the Louisiana Agriculture Center reported that 42,292 acres of Clearfield XL 745 rice were planted and accounted for 9.7% of rice crops in Louisiana. In addition to producing grain, these agronomic rice fields provide habitat and food for migrating waterfowl.

By planting multiple hybrid-rice varieties, farmers have a better chance to increase yield, quality, and profit. Hybrid rice has been designed to combat invasive weeds such as red rice (*Oryza sativa* var.) and to be moderately resistant to sheath blight, blast, and straighthead (Deliberto and Salassi, 2010). Hybrid rice is known to be a more abrasive or hairy rice than conventional rice varieties (Mayer, 2012). The abrasiveness has been known to cause faster wear on milling equipment but it has unknown effects on waterfowl. Waterfowl are known to consume hybrid waste grain or waste rice (rice not picked up or spilled in the harvest and transportation process). Waste rice is a high-energy food source that can be easily and quickly consumed by waterfowl (Ringelman, 1990).

The diet preference of waterfowl, however, may be controlled by many factors, including negative energy balances, hunting or dominance pressures, digestion inhibitors, blood-glucose levels, levels of swelling in the digestive tract, or, for pen-reared ducks, relative distance of the pans from the pen door (Duke, 1986; Dabbert and Martin, 1994).

Agricultural fields, especially rice fields, provide an important economic benefit to Louisiana in the form of hunting revenue. By leasing agricultural fields that waterfowl utilize for food sources and habitat, the landowner can add a revenue source. In 2017, there were over 46,900 × (1 ± 12%) active duck hunters and 1,083,900 × (1 ± 18%) ducks harvested in Louisiana (Raftovich et al., 2018). Gross farm value from hunting leases for waterfowl in Louisiana from 2017 to 2018 was $52.4 million (LSU AgCenter Staff, 2018). The 2011 National Survey of Fishing, Hunting, and Wildlife-Associated Recreation stated for Louisiana that trip- and equipment-related expenditures for waterfowl hunting generated about $3.0 billion in total economic output (Cabrera et al., 2015). Jeopardizing either of these (rice/hunting) enterprises would be economically damaging to the farmer.

A broiler chick's diet strongly influences the anatomy and physiology of its gastrointestinal tract (Frikha et al., 2009; Mateos et al., 2012). Poultry and waterfowl have similar gastrointestinal tracts, and many studies conducted on digestive organs focus on poultry (Gabriel et al., 2008; Frikha et al., 2009; de Verdal et al., 2011). Two studies, by de Verdal et al. (2011) and by Nir et al. (1994), concluded that a coarser diet would lead to a larger, better-developed gizzard and a lighter intestine, leading to more efficient digestion of starches and proteins. One of the few pen waterfowl studies showed that a well-developed gizzard actually improves gut motility (Lu et al., 2011). A well-developed gizzard improved performance, digestion, and energy use by effectively breaking down feed and allowing for quick transportation of nutrients to the pancreatic enzyme (Lu et al., 2011). The gastrointestinal tract influences ingestion, digestion, and absorption; any problems in digestion may partially influence growth, survival, or reproduction (Lu et al., 2011). Since hybrid rice is known to be coarser and more abrasive than conventional varieties, consumption by poultry and waterfowl could lead to a better developed digestive system. By having a coarser diet, waterfowl may have an increase in gizzard size, which has been shown to have beneficial effects on overall health in some waterfowl and poultry. Also, if the rice variety attracts or repels the waterfowl from certain fields, this could factor into a farmer's seed selection process.

Thus, the following experiments were conducted to determine if hybrid rice affected the growth and development of waterfowl and chickens.

## 2 Materials and methods

All experiments were approved by the McNeese State University Animal Use and Care Committee. Three experiments (Exp. 1, *n* = 144 broilers; Exp. 2, *n* = 118 ducklings; Exp. 3, *n* = 75 mature ducks) were conducted to evaluate the effect of rice variety on chicken, duckling, and duck growth and development. Average initial weights of the chicks and ducks were 34.8, 31.8, and 821 g in Exp. 1, 2, and 3, respectively. Average final BW of the chicks, ducklings, and ducks were 362.1, 446.6, and 1,202.0 g in Exp. 1, 2, and 3, respectively. In Exp. 1 and 2, hatchling chickens (Ross × Ross) and ducklings (mallards) were housed at the McNeese State University School of Agricultural Sciences Poultry Laboratory in Lake Charles, Louisiana. For Exp. 1 and 2, birds were maintained in environmentally controlled (32 °C) Petersime (Petersime Incubator Co., Gettysburg, OH) battery pens (33 cm × 99 cm) with raised wire floors. Continuous fluorescent lighting was provided for the duration of all experiments. All cages and dropping pans were cleaned daily.

In Exp. 3, hatchling mallard ducks (*n* = 100) were purchased (Metzer Farms, Gonzales, CA) and reared on a commercial duck farm (Chappapeela Farm, Husser, LA) for 30 d and then transported to the McNeese State University Farm in Lake Charles, Louisiana, where they were housed in an open-sided ventilated barn, in pens measuring 110 cm × 170 cm with tri-bar flooring. Diets were formulated to meet or exceed nutritional requirements (Table 1) for hatchling chicks (Exp. 1), ducklings (Exp. 2), and ducks (Exp. 3) (NRC 1994). The treatments consisted of 3 levels of unmilled Clearfield XL 745 rice (0%, 5%, or 10%). Chicks, ducklings, and ducks were allotted to dietary treatments: 8 replications of 6 broilers (Exp. 1), 8 replications of 4 or 5 ducklings (Exp. 2), and 3 replications of 8 or 9 ducks (Exp. 3). Treatment diets in mash form and water were provided ad libitum throughout the experiment. Average daily gain (ADG), average daily feed intake (ADFI), and gain-to-feed ratio (G:F) were determined weekly and for the entire experimental period (d 11, 14, and 35 in Exp. 1, 2, and 3, respectively).

**Table 1:** Diet composition (%, as-fed basis).

Item	Hybrid rice		
	0	5%	10%
Ingredients			
Corn	49.73	44.35	38.96
Soybean meal	40.12	40.17	40.22
Vegetable oil	5.44	5.77	6.10
Hybrid rice^1^	0.00	5.00	10.00
Calcium phosphate	1.49	1.49	1.49
Limestone	1.43	1.43	1.43
Salt	0.50	0.50	0.50
dl-methionine	0.38	0.38	0.39
Biolys^2^	0.30	0.30	0.30
Minerals^3^	0.25	0.25	0.25
Vitamins^4^	0.25	0.25	0.25
Threonine	0.06	0.06	0.06
Choline chloride^5^	0.05	0.05	0.05
Calculated composition			
ME, kcal/kg	3,200	3,200	3,200
Crude protein	23.20	23.17	23.14
Lysine	1.43	1.43	1.43
Calcium	1.00	1.00	1.00
Phosphorus	0.73	0.73	0.73

1 Unmilled Clearfield XL 745 rice (ME 2,940 kcal/kg, 7.3% crude protein, 0.24% Lys).

2 Contained 50.7% Lys∙SO_4_ (Evonik-Degussa Corp, Kennasaw, GA).

3 The vitamin premix used provided per kilogram of diet: vitamin A (vitamin A palmitate), 4,500 IU; vitamin D_3_,450 IU; vitamin E (vitamin E acetate), 50 IU; vitamin K (menadione sodium bisulfite), 1.5 mg; vitamin B_12_, 0.02 mg; vitamin H (d-biotin), 0.6 mg; folacin (folic acid), 6 mg; niacin, 50 mg; vitamin B_1_, 13.4 mg.

4 The mineral premix used provided per kilogram of diet: copper (CuSO_4_∙5H_2_O), 4.0 mg; iodine (KIO₃), 1.0 mg; iron (FeSO_4_∙7H_2_O), 60 mg; manganese (MnSO_4_∙H_2_O), 60 mg; selenium (Na_2_SeO_3_), 0.1 mg; zinc (ZnSO_4_∙7H_2_O), 44 mg; calcium (CaCO_3_), 723 mg.

5 It contains 600,000 mg/kg of choline.

After a 24-h feed withdrawal, birds were euthanized via carbon dioxide and placed in labeled Whirl-Pak bags for further dissection and analyses. In Exp. 1, 24 birds were euthanized at d 11. In Exp. 2, a total of 118 birds were euthanized (*n* = 24 at d 14 and 21) and *n* = 70 at d 28. In Exp. 3, 75 birds were euthanized at d 35. All samples were stored for 14 d at 3 °C until dissection and evaluation. At a later date, bird carcasses were allowed to warm to room temperature, and then gastrointestinal tracts (crop [broilers only], esophagus, proventriculus, duodenum, jejunum, ileum) and organs (heart, gizzard, liver, pancreas) were dissected and cleaned of digesta for linear measurements and weights.

Growth data were analyzed by analysis of variance (ANOVA) procedures appropriate for a completely randomized design (Exp. 1) using the GLM procedures of SAS (SAS Inst. Inc., Cary, NC) at the significance level (*P* < 0.05). In Exp. 2 and 3, growth data were analyzed using and randomized complete block design in a one-way ANOVA in the SPSS program at the significance level (*P* < 0.05). Tukey's Honestly Significant Difference (HSD) test was used to find difference among treatments, and significance was considered at *P* < 0.05.

## 3 Results

In Exp. 1, birds fed 10% rice experienced slower growth (*P* < 0.05) than birds fed the basal diet from 0 to 4 d and 0 to 7 d (Table 2). However, when compared to birds fed 5% rice, birds receiving 10% rice had an increased (*P* < 0.05) ADG from d 7 to 11 and overall (d 0 to 11). Average daily feed intake was greater (*P* < 0.05) in birds fed the basal and 10% rice diets from d 0 to 7, d 7 to 11, and d 0 to11. Birds fed diets containing 10% rice were less efficient (*P* < 0.05) from d 0 to 7.

**Table 2:** Growth performance of broilers.^1^

Item	Hybrid rice			SEM
	0	5%	10%	
Day 0 to 4				
ADG, g	11.2^a^	10.9^a,b^	10.4^b^	0.2
ADFI, g	18.2	17.9	18.5	0.3
G:F, g/kg	616.0^a^	612.0^a^	565.0^b^	14.0
Day 0 to 7				
ADG, g	15.7^a^	14.7^b^	14.9^b^	0.2
ADFI, g	20.3^a^	19.7^b^	20.7^a^	0.3
G:F, g/kg	773.0	751.0	724.0	10.0
Day 7 to 11				
ADG, g	23.5^a^	23.1^a^	24.7^b^	0.3
ADFI, g	33.6^a^	31.7^b^	33.3^a^	0.7
G:F, g/kg	701.0^a^	730.0^ab^	746.0^b^	15.0
Day 0 to 11				
ADG, g	20.7^a^	19.8^b^	20.8^a^	0.2
ADFI, g	24.8^a^	23.5^b^	24.4^a^	0.4
G:F, g/kg	835.0	845.0	852.0	11.0

ADG = average daily gain; ADFI = average daily feed intake; G:F = gain-to-feed ratio.

^a, b^ Within a row, means with different superscripts differ significantly (*P* < 0.05).

1 Data are means of 8 replications of 6 birds per replication with an initial and final BW of 34.8 and 362.1 g, respectively.

**Fig. 1:**
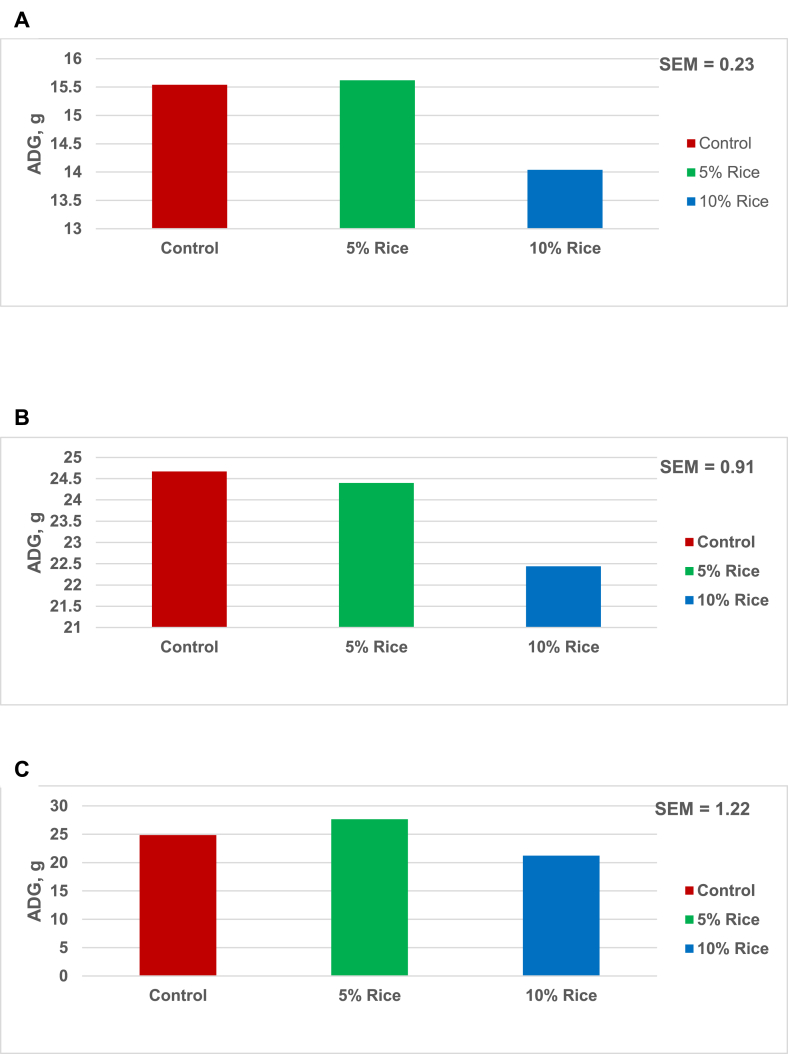
Average daily gain (ADG) of hatchling ducks from 0 to 7 d (A), 7 to14 d (B), 14 to 21 d (C) of age in Exp. 2. The ADG data are means of 8 replications of 4 or 5 ducks per replication with an initial and final BW of 31.8 and 446.6 g, respectively. The inclusion of 10% hybrid rice decreases ADG (*P* < 0.05) in (A) and (B).

**Fig. 2:**
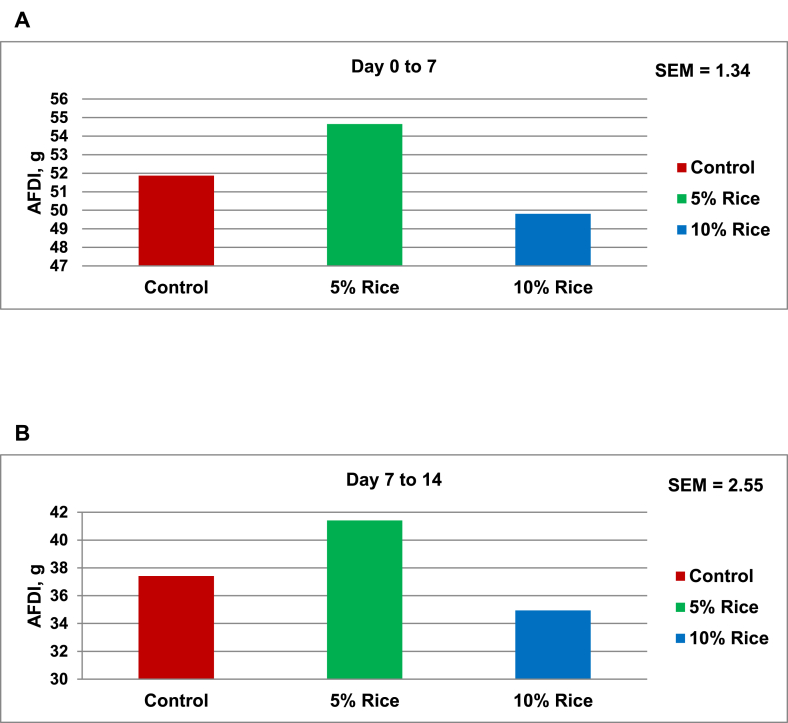
Average daily feed intake (AFDI) of hatchling ducks from 0 to 7 d (A), 7 to 14 d (B) of age in Exp. 2. The data are means of 8 replications of 4 or 5 ducks per replication with an initial and final BW of 31.8 and 446.6 g. The inclusion of 10% hybrid rice decreases (*P* < 0.05) ADFI in (A) and (B).

In Exp. 2, there was no difference (*P* > 0.10) in ADG between birds fed the basal diet and 5% rice during all growth phases (Fig. 1). However, 10% rice decreased (*P* < 0.05) growth and ADFI during the same periods (Fig. 2). Feed intake data were not reported past 14 d and growth data past 21 d because of bird removal for intestinal tract and organ analyses.

In Exp. 3, there was no difference (*P* > 0.10) for ADG, ADFI, and feed efficiency at any time in the experiment for birds receiving 0 or 5% rice (Table 3). In week 5, ducks receiving 10% rice had a lower feed efficiency (*P* < 0.05) compared to ducks fed the basal diet.

**Table 3:** Growth performance of mature ducks.^1^

Item	Hybrid rice			SEM
	0	5%	10%	
Day 0 to 7				
ADG, g	26.60	26.81	28.05	1.80
ADFI, g	101.07	102.93	100.62	12.93
G:F, g/kg	263.00	260.00	279.00	9.40
Day 7 to 14				
ADG, g	9.46	11.11	12.58	2.09
ADFI, g	99.99	111.04	107.19	10.34
G:F, g/kg	87.00	107.00	117.00	19.00
Day 14 to 28				
ADG, g	7.80	7.73	6.41	0.84
ADFI, g	103.04	102.03	105.44	2.13
G:F, g/kg	77.28	75.86	61.07	9.12
Day 28 to 35				
ADG, g	2.42	1.58	1.58	1.85
ADFI, g	97.73	100.68	102.12	6.45
G:F, g/kg	24.00^a^	16.00^ab^	9.00^b^	5.00
Day 0 to 35				
ADG, g	10.58	10.73	10.74	0.50
ADFI, g	47.12	48.41	46.61	0.90
G:F, g/kg	194.00	193.00	195.00	10.00

ADG = average daily gain; ADFI = average daily feed intake; G:F = gain-to-feed ratio.

^a, b^ Within a row, means with different superscripts differ significantly (*P* < 0.05).

1 Data are means of 3 replications of 8 or 9 ducks per replication with an initial and final BW of 821 and 1,202.0 g, respectively.

In Exp. 1, there was no difference (*P* > 0.10) in linear measurements (Table 4) or organ weights (Table 5). In Exp. 2, there was no difference (*P* > 0.10) in linear measurements (Table 6, Table 7). However, birds fed 10% rice had shorter (*P* < 0.05) ileum lengths than birds fed 5% rice when harvested at d 28 (Table 8). Organ weights were not different at d 14 or 21 (Table 9, Table 10). Ducks fed 5% rice had heavier (*P* < 0.05) pancreas and ileum weights when harvested at d 28 (Table 11). Jejunum weights of ducks fed 10% rice were lighter (*P* < 0.05) compared to ducks consuming diets with 5% rice.

**Table 4:** Broiler organ lengths (cm) at 11 d of age (*n* = 24).^1^

Organ	Hybrid rice			SEM
	0	5%	10%	
Esophagus	8.0	8.5	8.7	0.7
Duodenum	18.7	20.9	19.9	1.4
Jejunum	41.5	45.1	43.4	3.5
Ileum	42.7	47.2	42.4	3.8

1 One bird per pen was randomly selected for gastrointestinal tract evaluation. Data are means of 3 birds per treatment. No difference (*P* > 0.10).

**Table 5:** Broiler organ weights (g) at 11 d of age (*n* = 24).^1^

Organ	Hybrid rice			SEM
	0	5%	10%	
Liver	13.0	13.0	12.4	1.1
Heart	16.5	17.5	18.5	0.2
Gizzard	2.8	3.1	3.4	1.2
Crop	2.2	2.7	2.5	0.2
Pancreas	1.3	1.5	1.4	0.1
Esophagus	1.4	1.1	1.1	0.2
Proventriculus	3.0	2.7	2.7	0.2
Duodenum	4.0	4.0	3.3	0.6
Jejunum	7.1	7.4	6.6	0.7
Ileum	7.3	5.8	4.7	1.2

1 One bird per pen was randomly selected for gastrointestinal tract evaluation. Data are means of 3 birds per treatment. No difference (*P* > 0.10).

**Table 6:** Duckling organ lengths (cm) at 14 d of age (*n* = 24).^1^

Organ	Hybrid rice			SEM
	0	5%	10%	
Esophagus	12.8	13.3	13.0	0.6
Duodenum	19.8	19.4	20.5	1.1
Jejunum	45.3	45.8	44.3	1.6
Ileum	41.1	43.3	41.7	2.0

1 One bird per treatment was randomly selected for gastrointestinal tract evaluation. Data are means of 3 birds per treatment. No difference (*P* > 0.10).

**Table 7:** Duckling organ lengths (cm) at 21 d of age (*n* = 24).^1^

Organ	Hybrid rice			SEM
	0	5%	10%	
Esophagus	15.1	16.6	15.6	0.9
Duodenum	21.9	23.6	21.9	1.4
Jejunum	50.3	51.6	47.3	2.0
Ileum	47.7	48.2	45.7	2.2

1 One bird per treatment was randomly selected for gastrointestinal tract evaluation. Data are means of 3 birds per treatment. No difference (*P* > 0.10).

**Table 8:** Duckling organ lengths (cm) at 28 d of age (*n* = 70).^1^

Organ	Hybrid rice			SEM
	0	5%	10%	
Esophagus	17.3	17.5	17.7	0.5
Duodenum	24.3	23.7	23.3	1.0
Jejunum	49.8	53.2	49.9	1.8
Ileum	47.5^a,b^	50.5^a^	45.7^b^	1.8

^a,b^ Within a row, means with different superscripts differ significantly (*P* < 0.05).

1 Data are means of 8 replicates of 3 or 4 birds per pen.

**Table 9:** Duckling organ weights (g) at 14 d of age (*n* = 24).^1^

Organ	Hybrid rice			SEM
	0	5%	10%	
Liver	11.4	11.5	11.6	1.6
Heart	3.0	3.0	2.5	0.4
Gizzard	18.8	18.9	18.6	1.5
Pancreas	1.0	1.0	0.9	0.1
Esophagus	3.3	3.5	3.3	0.5
Proventriculus	2.3	2.5	3.2	0.8
Duodenum	4.8	4.1	4.5	0.5
Jejunum	6.1	7.1	6.0	0.7
Ileum	6.7	7.5	6.7	1.1

1 One bird per pen was randomly selected for gastrointestinal tract evaluation. Data are means of 3 birds per treatment. No difference (*P* > 0.10).

**Table 10:** Duckling organ weights (g) at 21 d of age (*n* = 24).^1^

Organ	Hybrid rice			SEM
	0	5%	10%	
Liver	15.6	14.9	13.1	1.4
Heart	4.7	4.5	3.8	0.4
Gizzard	28.9	28.9	26.1	2.2
Pancreas	1.0	1.0	1.0	0.02
Esophagus	4.3	6.5	5.0	0.6
Proventriculus	3.6	3.8	2.8	0.5
Duodenum	6.6	6.4	5.5	0.5
Jejunum	8.7	9.3	7.8	1.0
Ileum	9.1	10.4	8.1	1.2

1 One bird per pen was randomly selected for gastrointestinal tract evaluation. Data are means of 3 birds per treatment. No difference (*P* > 0.10).

**Table 11:** Duckling organ weights (g) at 28 d of age (*n* = 70).^1^

Organ	Hybrid rice			SEM
	0	5%	10%	
Liver	19.0	18.8	16.8	1.2
Heart	5.8	6.0	5.3	0.4
Gizzard	30.3	31.3	28.8	1.6
Pancreas	1.1^a^	1.3^b^	1.1^a^	0.1
Esophagus	4.1	4.1	5.4	1.4
Proventriculus	3.6	3.4	3.4	0.3
Duodenum	6.7	6.9	6.3	0.4
Jejunum	8.5^a,b^	9.7^a^	8.1^b^	0.6
Ileum	8.8^a,c^	10.5^b^	8.4^c^	0.7

^a,b,c^ Within a row, means with different superscripts differ significantly (*P* < 0.05).

1 Data are means of 8 replicates of 3 or 4 birds per replicate.

In Exp. 3, ducks fed 10% rice had heavier (*P* < 0.05) livers than ducks fed the basal diet (Table 12). There was no difference (*P* < 0.05) in organ length between treatments (Table 13).

**Table 12:** Mature duck organ weights (g) at 65 d of age (*n* = 75).^1^

Organ	Hybrid rice			SEM
	0	5%	10%	
Gizzard	43.8	45.4	46.0	1.8
Liver	24.6^a^	26.3^a,b^	27.7^b^	1.2
Ileum	12.2	13.0	13.1	1.0
Jejunum	12.1	12.6	12.0	0.8
Heart	11.4	11.9	12.0	0.5
Esophagus	6.7	7.5	6.3	0.7
Duodenum	5.0	5.5	5.2	0.4
Proventriculus	5.0	4.5	4.6	0.5
Pancreas	1.0	1.0	1.0	0.0

^a,b^ Within a row, means with different superscripts differ significantly (*P* < 0.05).

1 Data are means of 3 replicates of 8 or 9 ducks per replicate.

**Table 13:** Mature duck organ lengths (cm) at 65 d of age (*n* = 75).^1^

Organ	Hybrid rice			SEM
	0	5%	10%	
Esophagus	23.4	24.2	23.9	0.6
Duodenum	23.9	24.5	24.4	0.9
Jejunum	57.0	57.7	58.2	1.6
Ileum	53.0	53.7	54.9	1.7

1 Data are means of 3 replicates of 8 or 9 ducks per replicate. No difference (*P* > 0.10).

## 4 Discussion

This study investigated the effects of feeding unmilled rice at 3 levels in growing and adult broilers, ducklings, and ducks. Anecdotal evidence suggests that hybrid rice varieties which are planted to resist pests and weed pressure may reduce feed palatability and thus feed intake of waterfowl that winter in or near rice fields. Because rice is an important human food staple, most animal feeding studies focus on feeding byproducts of rice (e.g., bran, straw, hulls), and limited research has been conducted on feeding whole or ground rice to animals. Cromwell et al. (2005) found no adverse effects when pigs were fed ground glufosinate herbicide–tolerant rice or conventional rice to pigs. Our study found that, regardless of rice inclusion, birds (broilers, ducklings, and ducks) grew at a similar rate and that there were no detrimental effects from feeding 10% rice. Even though ducklings fed 10% rice had a reduced average daily growth, there were no differences in the ducks’ ultimate overall growth. A potentially limiting factor for feed consumption may be the unhulled seed or whole grain. However, if the unhulled rice is ingested, it may stimulate an increase in pregastric gizzard development that would be more efficient in grinding the whole grains. Previous studies have reported an increase in gizzard and duodenum weight when geese were fed whole-corn diets at 28 and 70 d of age (Lu et al., 2011). When mature ducks (30 d of age) were fed 5% or 10% unmilled rice for 36 d, organ length and weight tended to be numerically greater than the organ length and weight of ducks fed diets without rice. When ducklings (0 to 28 d of age) were fed 5% or 10% rice, there was no consistent pattern to organ length and weight compared to those of birds fed diets without rice. There was a numerical trend for longer and heavier organs of ducks receiving 5% rice diets. When ducklings received 10% rice diets, the organ lengths and weights tended to be less than those of ducklings on the other 2 diets. This may be due to the refusal or lower feed intake of the 10% rice diet. The contrasting organ data between the younger and older ducks in our study may suggest that unhulled rice may be detrimental to juvenile birds in percentages greater than 5% of the diet but when included at 5% or greater in diets of ducks 30 d of age or older have a beneficial or no effect on the digestive system.

## 5 Conclusion

The purpose of this study was to determine the effects of hybrid rice varieties on the growth performance and digestive tract development of broilers, ducklings, and mature ducks. Growth performance and intestinal tract development were similar in broilers regardless of percentage of rice included in the diet. When ducklings (0 to 28 d of age) were fed diets containing 10% rice, growth and feed intake were decreased. However, birds receiving the 5% rice diet had increased growth and feed intake when compared to birds fed the basal diet. Mature ducks (30 ± 2 d of age) had similar growth performance regardless of percentage of rice in the diet. Organ development (weights and lengths) differed when ducklings were fed 5% rice, but there was little effect in mature ducks. In Exp. 1 and 2, from 0 to 7 d, birds fed 10% rice had slower growth (*P* < 0.05) than birds fed the basal diet. In Exp. 3, the addition of rice did not affect growth, feed intake, or feed efficiency. The addition of 10% unmilled rice to broiler and duck diets may decrease growth performance.

## Author contributions

Frederick M. LeMieux: writing – review and editing, supervision. Courtney P. Villemarette: writing – original draft. Eddie K. Lyons: writing – original draft, supervision. Thomas H. Shields: writing – review and editing. Norman German: writing – review and editing.

## Conflict of interest

We declare that we have no financial or personal relationships with other people or organizations that might inappropriately influence our work, and there is no professional or other personal interest of any nature or kind in any product, service and/or company that could be construed as influencing the content of this paper.

## References

[bib1] Cabrera S., Aiken R., Fuller M. (2015). Economic impact of waterfowl hunting in the United States: addendum to the 2011 National Survey of Fishing, hunting, and Wildlife-associated Recreation Report 2011-6. http://file:///C:/Users/MSU/Downloads/2093.pdf.

[bib2] Cromwell G., Henry B., Scott L., Gerngross M., Dusek D., Fletcher D. (2005). Glufosinate herbicide–tolerant (LibertyLink) rice vs. conventional rice in diets for growing-finishing swine. J Anim Sci.

[bib3] Dabbert C., Martin T. (1994). Effects of diet composition and ambient temperature on food choice of captive mallards. SW Nat.

[bib4] de Verdal H.D., Narcy A., Bastianelli D., Chapuis H., Même N., Le Bihan-Duval E., Mignon-Grasteau S. (2011). Improving the efficiency of feed utilization in poultry by selection. 1. Genetic parameters of anatomy of the gastro-intestinal tract and digestive efficiency. BMC Genet.

[bib5] Deliberto M.A., Salassi M. (2010). Hybrid rice production costs and returns: comparisons with conventional & Clearfield varieties. LSU Ag Center Staff Report No. 2010-06.

[bib6] Duke G.E., Sturkie P.D. (1986). Alimentary canal: anatomy, regulation of feeding, and motility. Avian physiology.

[bib7] Frikha M., Safaa H., Serrano M., Arbe X., Mateos G. (2009). Influence of the main cereal and feed form of the diet on performance and digestive tract traits of brown-egg laying pullets. Poultry Sci.

[bib8] Gabriel I., Mallet S., Leconte M., Travel A., Lalles J. (2008). Effects of whole wheat feeding on the development of the digestive tract of broiler chickens. Anim Feed Sci Technol.

[bib9] LSU Ag Center Staff (2018). Louisiana summary agriculture & natural resources. https://www.lsuagcenter.com/%7E/media/system/7/9/6/7/796773af58d4c3e610063c7a8f7985f1/pub2382/ag/summary/2018_fullpdf.pdf.

[bib10] Lu J., Kong X., Wang Z., Yang Z., Zang K., Zou J. (2011). Influence of whole corn feeding on the performance, digestive tract development, and nutrient retention of geese. Poultry Sci.

[bib11] Mateos G.G., Jiménez-Moreno E., Serrano M., Lázaro R. (2012). Poultry response to high levels of dietary fiber sources varying in physical and chemical characteristics. J Appl Poultry Res.

[bib12] Mayer K. (2012). U.S. Long-grain rice industry: at a crucial crossroads. http://www.usriceproducers.com/files/398_2012.03.26_PentonWP.pdf.

[bib13] Nir I., Hillel R., Shefet G., Nitsan Z. (1994). Effect of grain particle size on performance. 2. Grain texture interactions. Poultry Sci.

[bib14] NRC (1994). Nutrient requirements of poultry.

[bib15] Raftovich R., Chandler S., Fleming K. (2018). Migratory bird hunting activity and harvest during the 2016-17 and 2017-18 hunting seasons.

[bib16] Ringelman J.K. (1990). Managing agriculture foods for waterfowl. United States department of the interior fish and Wildlife service. Fish Wildl Leafl.

